# Yoga as a treatment for chronic low back pain: A systematic review of the literature

**Published:** 2016-01-01

**Authors:** Douglas G. Chang, Jacquelyn A. Holt, Marisa Sklar, Erik J. Groessl

**Affiliations:** 1 Department of Orthopaedic Surgery, University of California, San Diego, USA; 2 VA San Diego Healthcare System, San Diego, USA; 3 Department of Psychiatry and Human Behavior, Warren Alpert Medical School of Brown University, Providence, USA

**Keywords:** Yoga, Low back pain, Complementary therapies, Muscle stretching exercises, Chronic pain

## Abstract

**Objectives:**

Chronic low back pain (CLBP) affects millions of people worldwide, and appears to be increasing in prevalence. It is associated not only with pain, but also with increased disability, psychological symptoms, and reduced quality of life. There are various treatment options for CLBP, but no single therapy stands out as being the most effective. In the past 10 years, yoga interventions have been studied as a CLBP treatment approach. The objective of this paper is to review the current literature supporting the efficacy of yoga for CLBP.

**Methods:**

A literature search through the beginning of 2015 was conducted in Pub Med for randomized control trials addressing treatment of CLBP with yoga.

**Results:**

In this review we evaluate the use of yoga as a treatment for CLBP. Specifically we evaluate how yoga impacts physical functioning and disability, pain, and associated psychological symptoms. We also evaluate possible mediators of the effect of yoga and the safety of yoga.

**Discussion:**

With few exceptions, previous studies and the recent randomized control trials (RCTs) indicate that yoga can reduce pain and disability, can be practiced safely, and is well received by participants. Some studies also indicate that yoga may improve psychological symptoms, but these effects are currently not as well established.

## Introduction

About one fourth of United States adults report low back pain, lasting a whole day or more, at some point within the past 3 months [1]. It is the most common cause of limited activity in people below the age of 45, is the second most frequent reason for visits to a physician, the third most common reason for surgery, and the fifth most common cause of hospital admission in the United States [2].

The majority of individuals with back pain and sciatica recover from an acute episode in 4–8 weeks [3–5]. 80–90% return to work within 12 weeks post injury [6]. However 25–80% of low back pain patients experience some form of recurrent back problem in the following year [4,5,7,8]. Among those who suffer from an episode of low back pain, one year later as many as 33% have moderate intensity pain, and 15% may have severe pain [7].

People suffering from chronic low back pain have other associated problems such as anxiety [9–11], depression [12,13], and disability [2,14], with a reduced quality of life [15,16]. Rates of major depression are 20% for persons with chronic back pain, compared to 6% for pain-free individuals [13].

The word “yoga” literally means “yoking”, or “joining together” for a harmonious relationship between body, mind and emotions to unite individual human spirit with divine spirit or the True Self [17,18]. Yoga involves a process of physical and mental training towards self-realization, the practice of which has eight component limbs. The eight components guide conduct within society, personal discipline, postures/poses (“asanas”), breathing, concentration, contemplation, meditation and absorption/stillness. As classically described, yoga poses comprise just one of the eight components of a broader discipline of physical, mental, and spiritual health. Modern Hatha yoga usually combines elements of postural positioning, breathing, concentration, and meditation. A typical Hatha yoga program involves a group led by an instructor for a ~ 60–90 minute session. The instructor provides guidance for correct postures, breathing and focus. They often encourage positive self-images. Iyengar yoga has a focus on holding postures, and the use of modifications (such as blocks, belts, chairs, blankets) to accommodate individual physical abilities. Other yoga styles exist and the experience in one style or class can be very different. The intensity can range from gentle to strenuous, with some types of yoga providing a cardiovascular workout, and others focused on relaxation and calmness. Another experiential factor comes from the yoga center itself, which can provide a sense of social and spiritual community.

Yoga popularity has grown tremendously in the past several years. National Health Interview Survey data conducted by the Centers for Disease Control and Prevention (CDC) show increased usage for complementary and alternative medicine (CAM) treatments [19]. In 2007, yoga was the 7^th^ most commonly used CAM therapy. CAM therapies are used mostly to treat musculoskeletal conditions, in particular back pain and to a lesser degree neck pain.

CLBP pain affects millions of people. There are many treatment options, but few have strong evidence for being effective [20,21]. Several randomized control trials (RCT) about yoga’s effect on low back pain have been completed; however they have varying outcome measures about pain and functional disability. A few meta-analysis studies were completed with 2011 searches, generally showing a positive effect, but limited in general by a relatively small total number of eligible RCTs [22–25].

Holtzman et al. conducted an electronic search in 2011 to identify 8 yoga articles, with a focus on pain and functional disability [23]. Ward et al. searched through different databases, also in 2011, to identify 17 articles on functional disability, pain and depression [24,25]. However the relevance and quality of the articles was limited. Of the 17 articles, 12 were focussed on back pain. These 12 included two pilot studies considered of poor methodological quality, and only three of the identified CLBP studies were considered to have an acceptable adherence to the intervention. Cramer et al. also searched through 2011 articles and focused on the outcomes of pain, disability, and quality of life. They used yet another statistical method to winnow a list of yoga and low back pain papers down to 8 studies [22]. Although the Holtzman et al., Ward et al. and Cramer et al. studies used very different search strategies, they all winnowed down their findings in different, semi-objective fashions to an essentially identical, smaller list of yoga studies for further consideration.

This report is unique with updates not available in the older review articles [21–25]. This paper reviews randomized control trials, as well as randomized studies, comparing yoga to current exercise interventions. In addition, this paper reviews the findings in the existing literature as they relate to physical functioning and disability, pain, and psychological factors, as well as a review of findings on the biological mechanisms of yoga on back pain.

## Materials and Methods

**Figure 1:**
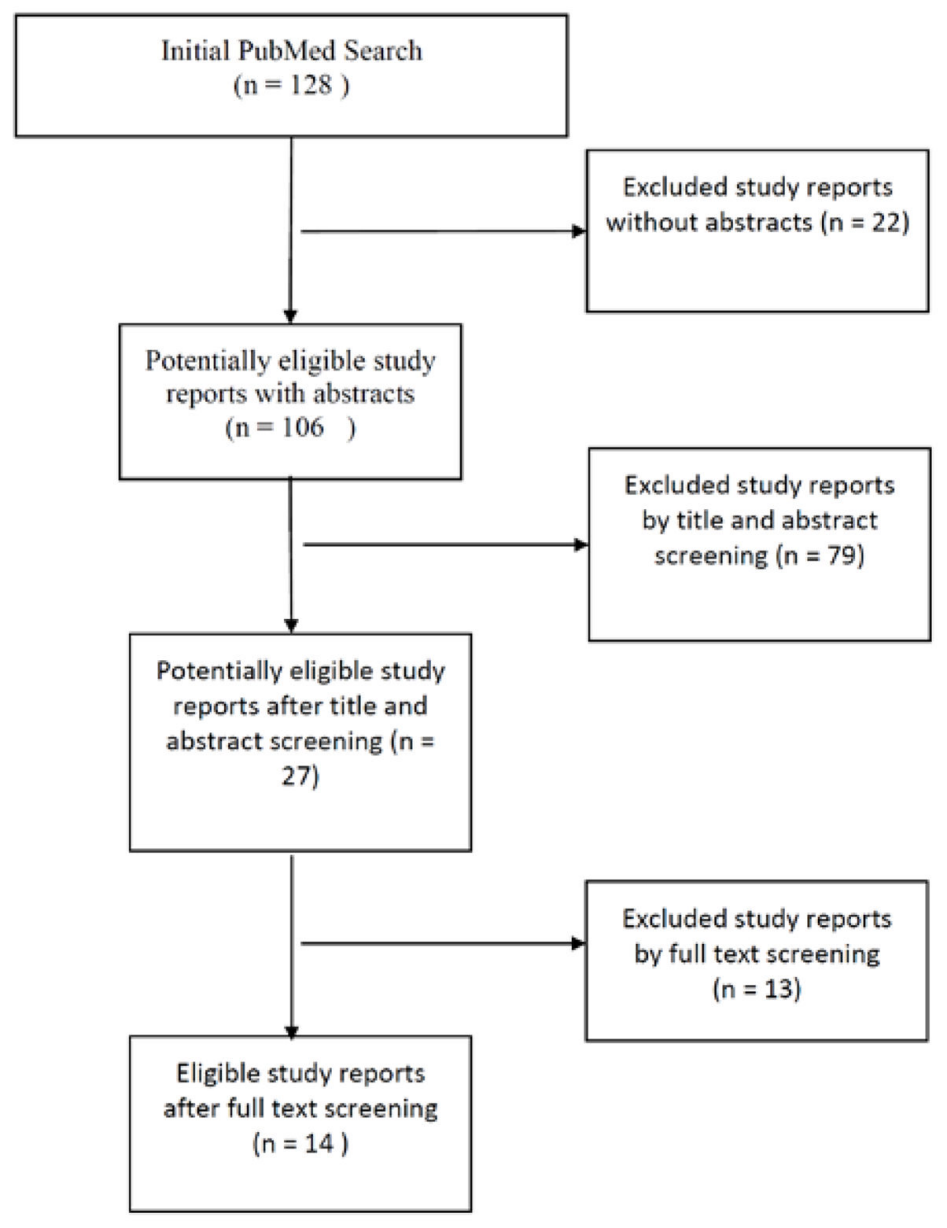
Flow chart representing the search and selection of articles for review.

A search in PubMed was conducted in the beginning of 2015 for randomized control trials of yoga and low back pain. The initial search of “yoga and back pain” identified 128 articles. Study reports without abstracts were excluded, returning 106 articles. Titles and abstracts were then screened for relevance to yoga and back pain, resulting in 27 articles (see Figure 1).

These articles underwent full text review. For this project, inclusion criteria included (1) yoga was actually studied as an intervention, (2) research subjects had medically diagnosed low back pain, (3) low back pain was chronic, (4) an original article, (5) a clinical trial, (6) study involved n≥20 subjects, (7) published in English. 14 study reports were determined to be eligible for review after this full text screening. These studies underwent extensive review and were rated using the Oxford Centre for Evidence Based Medicine 2011 Levels of Evidence criteria [26,27]. Most of the papers reviewed are level 2–4, with the majority being level 2 (Table 1). Due to the variety of outcome measures reported in the articles reviewed, we divided this paper into several outcome categories including: impact of yoga on physical functioning and disability, the impact of yoga on pain, psychological impacts of yoga, and biological mechanism of yoga on back pain.

## Results

### Physical functioning and disability

Yoga treatment studies of CLBP typically utilize some measure of physical functioning and disability as a primary outcome. Such outcomes can be tied to physiological performance, or validated questionnaires with specific behavioral items. Most yoga studies do demonstrate beneficial effects for adults suffering from CLBP (Table 1).

A small randomized controlled trial, pilot study demonstrated trends for the yoga group in terms of improved balance and flexibility, and decreased disability and depression [28]. However, the study was weakened by the small sample size (n=22 participants) and a high dropout rate among the control group. As such, no statistical significance was observed.

The impact of Iyengar yoga therapy was assessed in a 16-week, randomized controlled trial involving subjects with non-specific CLBP compared to an educational control group [29]. The yoga subjects had less pain (Short Form-McGill Pain Questionnaire) and lower functional disability (Pain Disability Index) than the controls. Unfortunately there was a 30% drop-out rate in this study. Williams et al. conducted another 24-week study that showed significantly greater reductions in functional disability (Oswestry Disability Index), pain intensity (Visual Analog Scale) and depression (Beck Depression Inventory-Second Edition) among the subjects randomized to the yoga intervention group [30]. Both of these studies were limited by a reliance on self-report measures, a relatively healthy study population, and lack of controls for attention and physical activity between the treatment and control groups [29].

80 patients with CLBP participated in an intensive seven day long, residential yoga program. The effect of yoga on disability (Oswestry Disability Index), quality of life (World Health Organization Quality of Life-BREF) and flexibility was studied [31,32]. The intervention group practiced daily meditation, yoga exercise, chanting and went to lectures. The control group followed a daily routine of exercise, non-yogic breathing exercises, educational lectures and additionally filled their time watching nature programs. This control is different than the usual or no care control used in other studies. There was a significant difference in disability between groups, with the yoga group experiencing a greater improvement than the control group. The yoga group showed a greater increase in flexibility and reduction in pain (section 1 of the Oswestry Disability Index) than the control group [31,32].

Predictors of outcome were studied in 53 adults who were already involved either with yoga or a physical therapy intervention to treat CLBP. No significant differences in disability (Roland-Morris Disability Questionnaire) were seen at 6 weeks [33]. A major finding in both groups was that back pain self-efficacy was the most important predictor of pain, health status and disability. Self-efficacy refers to an individual’s belief in the capacity to change outcomes through their own actions, and was measured by the Back Pain Self-Efficacy Scale. A significant limitation of this study is the presence of self-selection bias because the participants were already enrolled/self-selected into the yoga or physical therapy groups prior to the study.

The effects of yoga on balance and gait were studied in an 8 week pilot study involving adult women (n=27) with musculoskeletal problems, such as osteoarthritis and low back pain [34]. The subjects’ balance and gait parameters were statistically improved compared to the pre-study values. The small sample size and lack of control group in this study make it difficult to attribute the improvements exclusively to the yoga intervention. Additionally the study failed to describe the nature of the relatively young (age 30–45 yrs) subjects’ musculoskeletal problems in any detail.

The effect of yoga on physical functioning has been described in two large randomized trials [35,36]. Sherman et al. studied yoga compared to stretching or a self-care book approach for patients with chronic low back pain [35]. In all the groups, function and symptoms improved over time. The yoga and stretching groups reported similarly improved results compared to the self-care group. The authors concluded that yoga benefits are due mainly to the benefits of physically stretching and strengthening the body, and not due to yoga’s mental aspect.

Tilbrook et al. studied the long-term effectiveness of a 12-week yoga program versus a back pain education booklet for low back pain patients [36]. The yoga group had significantly better back function (Roland-Morris Disability Questionnaire) than the usual care group at 3, 6, and 12 months follow-up. This study did not see any difference between interventions, in a secondary outcome, the Aberdeen Back Pain Scale for health related function and pain [37,38]. However this scale is noted to have significant weaknesses and is not suggested anymore for use [39].

A randomized dosing trial compared once versus twice-weekly yoga classes for CLBP in predominantly low income, racially diverse and more severely impaired populations [40]. Subjects (n=95) completed a 12-week intervention. Pain and back-related function improved in both groups, with no difference between the once and twice-weekly groups. There were several study limitations including the inability to blind participants, the use of self-reported measures, lack of a non-yoga control group, differential adherence between groups, high use of non study treatments, and no long-term follow up. The results suggest in an underserved population, weekly yoga classes do not increase benefit and present more difficult compliance issues.

A four-week, randomized control trial of a virtual reality-based Wii yoga program versus physical therapy and trunk stabilizing exercise in 30 middle-aged women with low back pain was presented [41]. Significantly improved outcomes were observed in both groups, for pain (visual analog scale), function (Oswestry low-back pain Disability Index, Roland-Morris Disability Questionnaire), and fear (fear avoidance beliefs questionnaire) scores. There were financial and time benefits provided to the middle aged women by the home, virtual reality based program. Limitations of this study included the lack of a traditional yoga control group, and the lack of frequency and dosing of exercise information in the control group. Another limitation is the specific age range and gender of the subjects, but as discussed by the authors the target group is middle aged women who have significant demands on their time and money for housekeeping and childrearing activities.

### Pain

A number of studies demonstrate yoga’s effectiveness in reducing chronic low back pain. Williams et al. evaluated clinical levels of pain (using the Short Form-McGill Pain Questionnaire), pain-related fears to movement (Tampa Scale of Kinesiophobia), and pain beliefs (Survey of Pain Attitudes) [29]. The yoga intervention resulted in a two times greater reduction in pain, and reduced pain medication usage, compared to the educational control group. There was no significant difference in pain attitudes or movement fears, perhaps due to the study not having enough statistical power for these outcomes. Williams et al. further demonstrated the effectiveness of a 24 week Iyengar yoga program on improving chronic low back pain [30]. Individuals randomized to the yoga group showed greater improvements in pain intensity than in the control group.

A single group, pre-post study of military veterans who participated in a clinical yoga program at a large VA medical center showed improvement for pain between baseline and 10-weeks [42]. Pain was measured using a visual pain scale modification of the visual analog scale. Among the various indicators of the yoga “dosing” (i.e. amount of intervention), decreased pain was significantly correlated to the actual attendance. Additional analysis of this same study demonstrated that females experienced greater improvements in pain compared to the males [43]. The women had significantly greater improvements on depression, pain “on average”, energy, and Short-form 12 mental health. No gender difference was demonstrated for pain “at its worse”, total pain score, or Short-Form 12 physical health.

Saper et al. studied the effect of yoga on back pain among low income, racially diverse subjects [40]. The average low back pain intensity for the previous week (as measured by the Visual Analog Scale), was significantly reduced from 7 to 5 after the 12 week intervention, regardless of whether subjects attended once-or twice-weekly yoga classes [40].

Sherman et al. measured pain “bothersomeness” instead of pain severity because of the complex nature of pain [44]. Subjects rated their back pain during the previous week on an 11-point scale, from “not at all” to “extremely” bothersome, in response to a 12-week yoga, exercise or book education intervention. All interventions were helpful, but the yoga and exercise groups improved

Back pain “bothersomeness” was studied in individuals already involved either with yoga or a physical therapy intervention [33]. Both interventions helped, and there were no significant group differences between yoga and physical therapy after 6 weeks of treatment. However, the baseline characteristics of the yoga group were such that they demonstrated less back pain and disability to begin with.

A randomized control trial evaluated the impact of Iyengar yoga on pain intensity (Visual Analog Scale) and health related quality of life in subjects with nonspecific chronic low back pain [45]. The study compared yoga therapy to conventional exercise therapy, with 6 month follow up. Both interventions resulted in significant benefits, with the yoga intervention having the greater impact.

### Psychological impact of yoga

Yoga’s effect on psychological health has not been well characterized in the scientific literature to date. Galantino et al. conducted a pilot study of depression and a Hatha yoga intervention [28]. As mentioned, this study had a small sample size and a high dropout rate. They demonstrated a non-significant trend towards decreased depression in their yoga intervention group.

Groessl et al. studied the effect of yoga on depression and quality of life (Short-Form 12 version 2) in veterans with back pain [42]. They found significant improvements in depression, and a trend towards significant improvements for the Mental Health Scale of the SF-12. The improvement in depression tended to be associated with the subjects’ self-reported amount of home practice.

The impact of Iyengar yoga on depression (Beck Depression Inventory) was studied in subjects with CLBP [30]. The subjects randomized to the yoga group showed greater improvements in depression than those in the control group. One limit of the study was that yoga group received more attention than the self-directed control group. Another limit was the lack of controls for physical activity between the groups.

Tekur et al. found their yoga group experienced significantly greater improvements in the psychological subscale of their quality of life scale (World Health Organization Quality of Life-BREF) compared with the controls [32]. However, this residential-based study involved additional elements (i.e. 8 hours a day of interactive lectures, chanting, meditation sessions) than typical Hatha or Iyengar yoga interventions. Furthermore, this was only a short-term (i.e. 1 week follow up) study.

One randomized controlled trial [36] and an affiliated pilot study [46]studied yoga versus “usual care” with the mental health ShortForm 12. The pilot study found no significant difference, but it was under-powered (n =20) [46]. Their follow-up study was adequately powered. They still found no significant difference in mental health function at the 3-, 6- and 12 month assessments (although the 3- and6-month assessments demonstrated a trend towards improvement)[36].

### Biological mechanisms of yoga on back pain

A few studies have explored the mechanisms by which yoga might affect back pain. Sherman et al. and Lee et al. investigated several possible mediators, including serotonin, cortisol, dehydroepiandrosterone (DHEA), and brain derived neurotrophic factor (BDNF) [47,48]. Additionally Sherman et al. investigated psychological factors that may mediate the effect of yoga on back pain.

These factors included cognitive appraisal measures (fear avoidance, self-efficacy and self-awareness), affect and stress (psychological distress, perceived stress and positive states of mind), physical activity, and neuroendocrine function [47]. Neuroendocrine function was measured with cortisol and DHEA levels from saliva samples. The goal was to identify which measure had the biggest effect on back-related dysfunction (Roland-Morris Disability Questionnaire) in the yoga versus stretching versus self-care groups. Self-efficacy, and hours of back exercise were the most significant contributors to the effect of yoga. Sleep disturbance also played a small role. No effect was seen from cortisol or DHEA levels. Yoga and stretching had similar effects. One limit was the fairly healthy study population.

Lee et al. investigated the effect of yoga on pain, BDNF, and serotonin in premenopausal women with chronic low back pain [48]. The yoga group had decreased pain, increased BDNF and unchanged serotonin. The untreated control group had increased pain, decreased BDNF and decreased serotonin. This suggested that the beneficial effects of yoga are associated with elevated serum BDNF levels and maintained serotonin levels. There were several limitations including small sample size, gender bias, and a control group that could better account for group socialization effects. It’s also not clear why the control group exhibited increased pain after the intervention.

### Safety of yoga

Low back pain itself is a persistent condition with a known high rate of recurrence and a high rate of incomplete resolution [8,49]. Therefore in studying subjects with back pain, it would not be surprising to see occasional adverse events. Overall, however, it does not appear that yoga presents a significantly increased risk for normal individuals or patients with back pain. In one study, among 30 subjects randomized to yoga, one adverse event was reported [29]. That subject had symptomatic osteoarthritis and was diagnosed with a herniated disc during the study. Medical review of the adverse event by the Institutional Review Board determined it was unrelated to the yoga postures.

No serious adverse events were reported among 101 subjects in yoga versus exercise trial [44]. One yoga participant discontinued class due to migraine headaches, one exercise participant ‘strained’ her back and sought care from a chiropractor. More recently, Sherman et al. found an equal number of mild to moderate adverse events (mostly temporarily increased back pain) among the subjects in both the yoga and a more traditional stretching intervention [35]. One of the 87 yoga class subjects experienced a serious event, a herniated disc. One in the self care control group reported increased pain. Despites these adverse events, overall the yoga and stretching groups had moderately improved outcomes.

8% of 156 yoga participants and 1% of 157 usual care participants reports adverse events in the study by Tilbrook et al. [36]. In the yoga group, 11 events were nonserious and related to temporarily increased pain. 1 event was serious, and occurred in an individual with a history of severe pain after any physical activity. In the usual care group there was actually a death, and also an injury unrelated to the intervention.

In a comprehensive review of non-pharmacological and non-invasive therapies for chronic low back pain, there were discovered only rare reports of serious adverse events [50]. However, better reporting of harms was suggested as a need.

## Discussion

Low back pain risk factors include previous back pain episodes, high physical demands of work, low job satisfaction, age, back weakness, and smoking [51–53]. Care seeking and disability due to chronic low back pain depend more on psychosocial issues than on individual clinical features or workplace physical demands [53]. Identifying and addressing these psychosocial factors helps improve outcomes and limit costs [20].

Among the treatments for CLBP, there is variable evidence to support effectiveness of non-pharmacologic [50], and medication [54]management. Yoga, in comparison to spinal manipulation, physical therapy, and acupuncture, may be more cost effective because it can be delivered in a group format and self-administered at home. However, actual cost analysis of yoga interventions is needed.

This literature review suggests that yoga is effective in reducing pain and disability, and improving both physical and mental function. The Sherman et al. study employ used a three-arm intervention, and thus, provides important “comparative effectiveness” data by comparing yoga to a conventional stretching program led by physical therapists and a self-care book from primary care providers [35]. A few key points derive from their work, which is supported also by other research [33]. Yoga was not superior in effectiveness compared to conventional stretching. Self-efficacy and hours of back exercise may be the most important factors for both conventional exercise therapy and yoga.

To this point, compared to traditional exercise programs derived physical therapy, yoga could provide superior compliance and benefit in the long term. Yoga poses, once learned, might be more easily remembered by patients because the poses and their associated names tend to have universal recognition. Yoga programs, even ‘adaptive’ or ‘senior’ classes, are accessible in most cities, at studios, local gyms, recreation centers and hotel wellness centers. In contrast, patients finishing a physical therapy program may receive a variety of suggested exercises with no standardization, and little similar to what another physical therapist might dispense. Patients can lose, forget or not even receive a home exercise program from their therapists at the conclusion of a formal physical therapy prescription. Long term outcome studies are needed to explore this hypothesis because most yoga studies to date have lasted less than 26 weeks, with the exception of the study by Tilbrook et al. [36].

Yoga, with its spiritual and psychological underpinnings, potentially might provide greater mental health benefits compared to traditional physical therapy. However, the impact of yoga on depression has been evaluated in only a small number of studies. Significant effects on depression were found in two studies [30,42], but only non-significant trends were found for the Mental Health Scale SF-12 [42]. Sherman et al. found that self-efficacy and also sleep were important psychological benefits of yoga on low back pain [47]. With the high rates of depression among sufferers of CLBP, further research in this area is needed [20]. To what extent yoga may impact other mental health conditions (e.g. anxiety disorders, which are not as well measured by the SF-12) is also an important research direction.

Lastly, safety data from the largest and most recent trials suggest about a 10–15% incidence of temporarily increased low back pain, and two identified cases of herniated disc. It appears that yoga participation is not without risks. However, the great majority of participants do appear to experience considerable benefits without many problems.

Overall, yoga is an intervention which appears to be well positioned, as the healthcare system shifts from caring mostly for patients with acute illness to caring mostly for patients with chronic disease, and where healthcare providers seek to design preventative strategies against the chronic conditions of modern society. More specifically, because it is a reflective activity, yoga may find particular application among military veterans who must live with the long term effects of wartime trauma [55].

## Conclusion

Yoga appears as effective as other non-pharmacologic treatments in reducing the functional disability of back pain. It appears to be more effective in reducing pain severity or “bothersomeness” of CLBP when compared to usual care or no care. Yoga may have a positive effect on depression and other psychological co-morbidities, with maintenance of serum BDNF and serotonin levels. Yoga appears to be an effective and safe intervention for chronic low back pain.

## Figures and Tables

**Table 1:** 

First Author (Year)	Study design	n	Yoga intervention	Comparison intervention	Primary outcomes	Main results	Oxford Level of Evidence
Evans et al. (2010)	Self selected treatment groups non-	53	Weekly yoga classes for 6 weeks, 120-min	Twice weekly individualized physical therapy sessions, 45–60 min	Pain medication use, Back pain bothersomeness, Back Pain Self-Efficacy Scale, Roland-Morris Disability Questionnaire, Short Form-36 health status, Treatment satisfaction	No significant group differences in treatment effect on pain and disability at 6 weeks. Self-efficacy was the most important predictor of pain, disability, and health status at 6 weeks for both groups. Self-efficacy was a stronger predictor of disability at 6 weeks for the physical therapy group.	4
Galantino et al. (2004)	RCT	22	Twice-weekly 60-min classes, 6 weeks	Waitlist control	Forward reach, Sit and reach, Oswestry Disability Index, Beck Depression Inventory	No significant intervention effects Limited sample size and dropout rate contribute to non-significance.	3
Groessl et al. (2008)	Single group, Pre-post	49, Veterans	Weekly yoga classes, attendance of at least 8 sessions over 10 weeks, home practice	n/a	Pain, Energy/fatigue, CESD-10, SF-12, attendance/home practice	Less pain, more energy, less depressive symptoms, and better HRQOL 10 weeks after starting the program. Greater attendance related to better outcomes. Frequency of home practice was associated with improved outcomes.	4
Kim et al. (2014)	RCT	30	30 min Wii based yoga program 12 session over 2 weeks	30 min trunk stabilizing exercise and 20 min standard physical therapy	VAS, pressure algometry, Oswestry low back pain disability index, Roland-Morris Disability Questionnaire, fear avoidance beliefs questionnaire	Both groups had significant improvement in all outcomes, with yoga group having more significant improvement.	2
Nambi et al. (2013)	RCT	120	Once weekly yoga class, home practice	Conventional exercise therapy	VAS, health related quality of life	Both groups had improvement in all outcomes, with the yoga group having a more significant improvement.	2
Saper et al. (2009)	RCT	30, Low income minorities	Weekly Hatha yoga classes, for 12 weeks, 75-min	Waitlist, usual care	Roland-Morris Disability Scale, pain	Yoga group reported greater decreases in pain, less analgesic use, less opiate use, and greater overall improvement than the usual care group. Reference [56].	3
Saper et al. (2013)	Dosing trial	95, Low income minorities	Once-weekly yoga, 60 min classes, 12 weeks	Twice-weekly yoga, 60 min, 12 weeks	Roland-Morris Disability Questionnaire, pain	No difference between once or twice weekly yoga class practice.	1
Sherman et al. (2011)	RCT	228	Weekly stretching classes for 12 weeks, 75 min, self-care book	Weekly stretching classes for 12 weeks, 75 min, Self-care book	Roland-Morris Disability Scale, bothersomeness	Similar effects of yoga and stretching.	2
Tekur et al. (2008)	RCT	80, India	Week-long intensive residential yoga program, standardized daily yoga practice	Week-long residential program, standardized daily non-yoga exercise and lectures on CLBP	Oswestry Disability Questionnaire, Spinal flexibility	Yoga group showed greater decrease in disability, and greater increase in spinal flexion, spinal extension, and left lateral flexion, than the control group	2
Tekur et al. (2010)	RCT	80, India	Week-long intensive residential yoga program, standardized daily yogic practices	Week-long residential program, standardized daily nonyogic exercises and lectures on CLBP	Perceived Stress Scale, WHOQO-BREF, Straight leg raising	Negative correlations between baseline stress and WHOQOL-BREF domains and total score. Greater improvements in WHOQOL-BREF domains for yoga group. Greater improvements in straight leg raising in the yoga group.	2
Tilbrook et al. (2011)	RCT	313, England	Weekly yoga classes for 12 weeks 75-min	Usual care	Roland-Morris Disability Scale,	Greater improvements for yoga group in back-function at 3,6, and 12-month follow-up. Greater improvements in the yoga group in Pain Self-Efficacy Questionnaire scores at 3, and 6 month follow-up	2
Ulger et al. (2011)	Single group, pre-post	27, female	Twice-weekly 60-min classes, 4 week	n/a	Static balance, Gait Parameters	Improvements on all gait and static balance parameters following yoga intervention. Limitations due to sample size and lack of control intervenient/group.	4
Williams et al. (2005)	RCT	60	Weekly yoga classes for 16 weeks, 90-min	Educational control group (weekly, newsletters, 2 lectures and handouts on chronic low back pain)	Pain (PDI, SF-MPQ, PPI, VAS), Pain-related fears (TSK), Pain attitudes (SOPA), Coping (CSQ-R), Self-efficacy (BPSES), Range of motion, Medication use, Adherence	Yoga group had less functional disability, two times greater reduction in pain, and a greater decrease in the use of pain medications than the control group No evidence for ta treatment effect on the psychological and behavioral subscales.	2
Williams et al. (2009)	RCT	90	24 weeks of twice-weekly 90-min yoga classes	Self-directed standard medical care	Oswestry Disability Questionnaire, Visual Analog Scale, Beck Depression Inventory, Medication use	Greater reductions on functional disability and pain intensity in the yoga group. Depression was significantly lower in the yoga group. No difference in medication use compared to other interventions.	2
